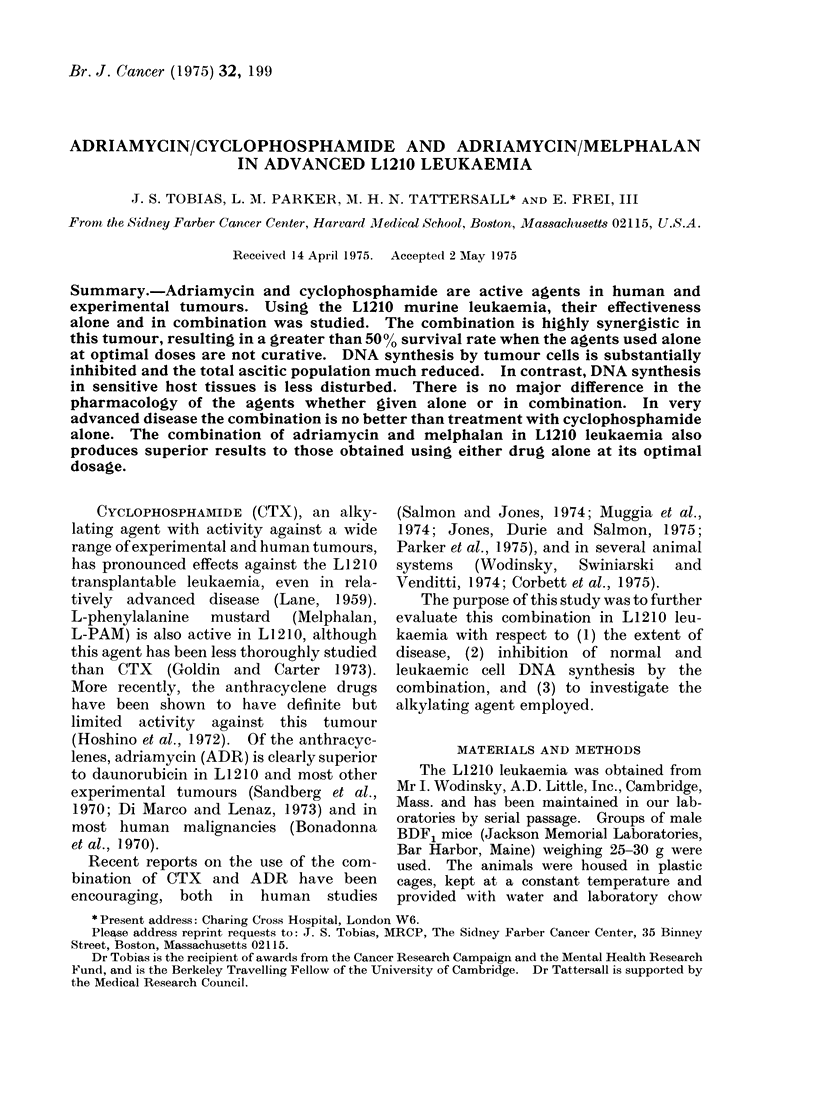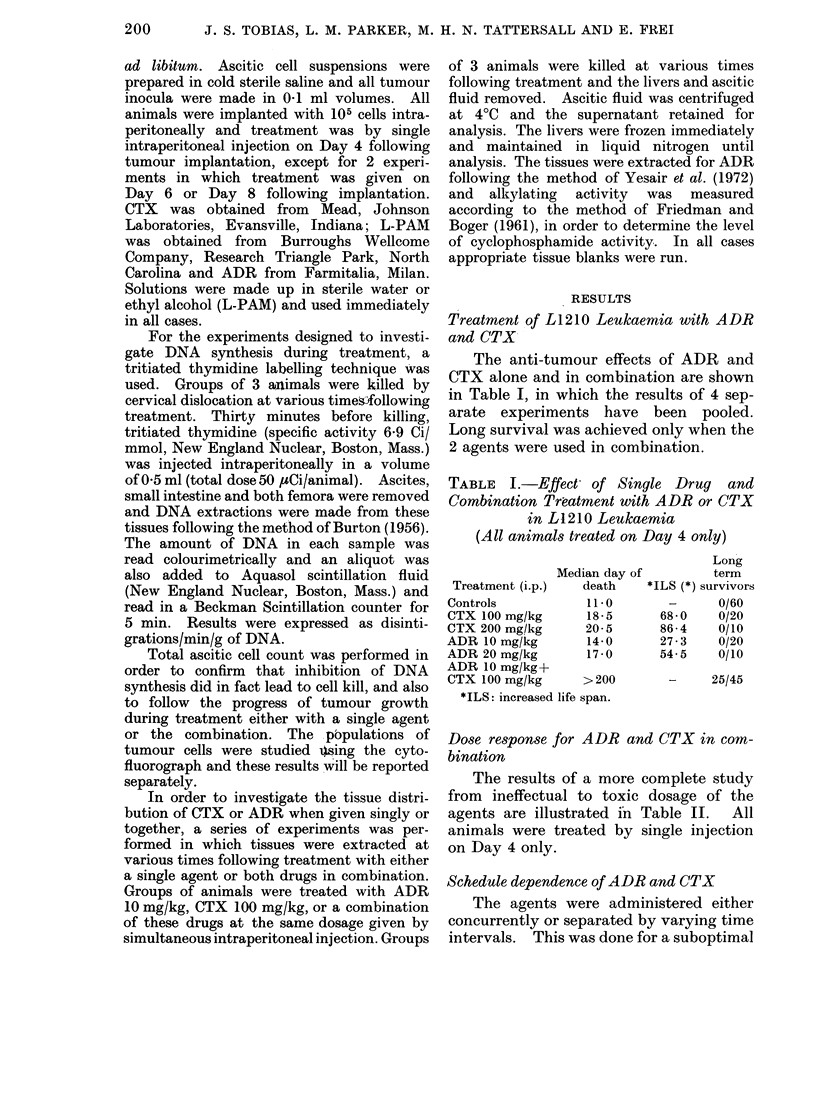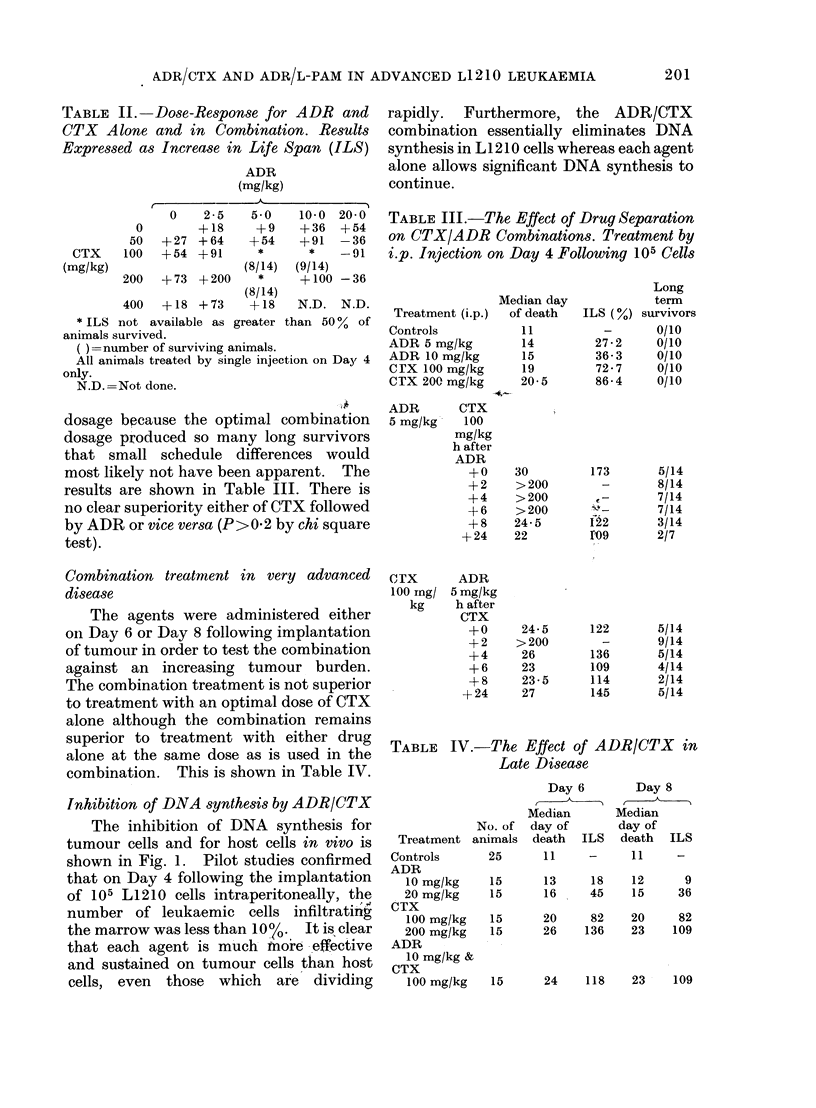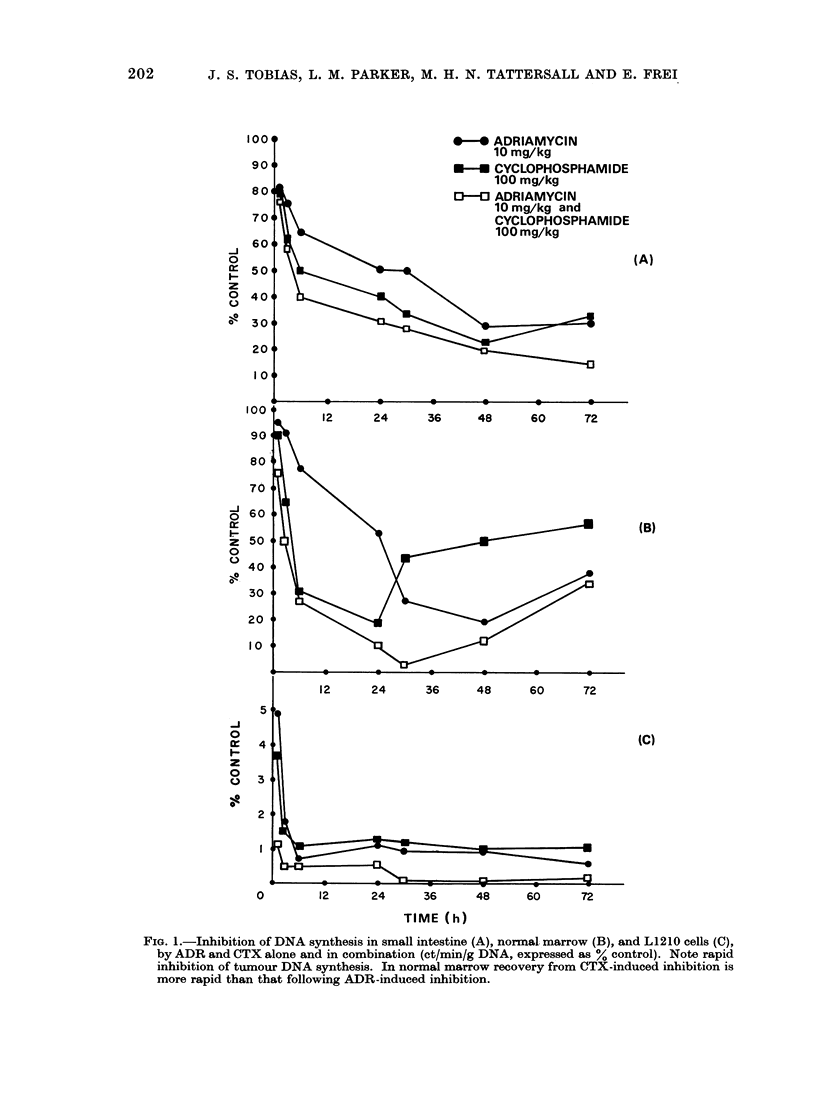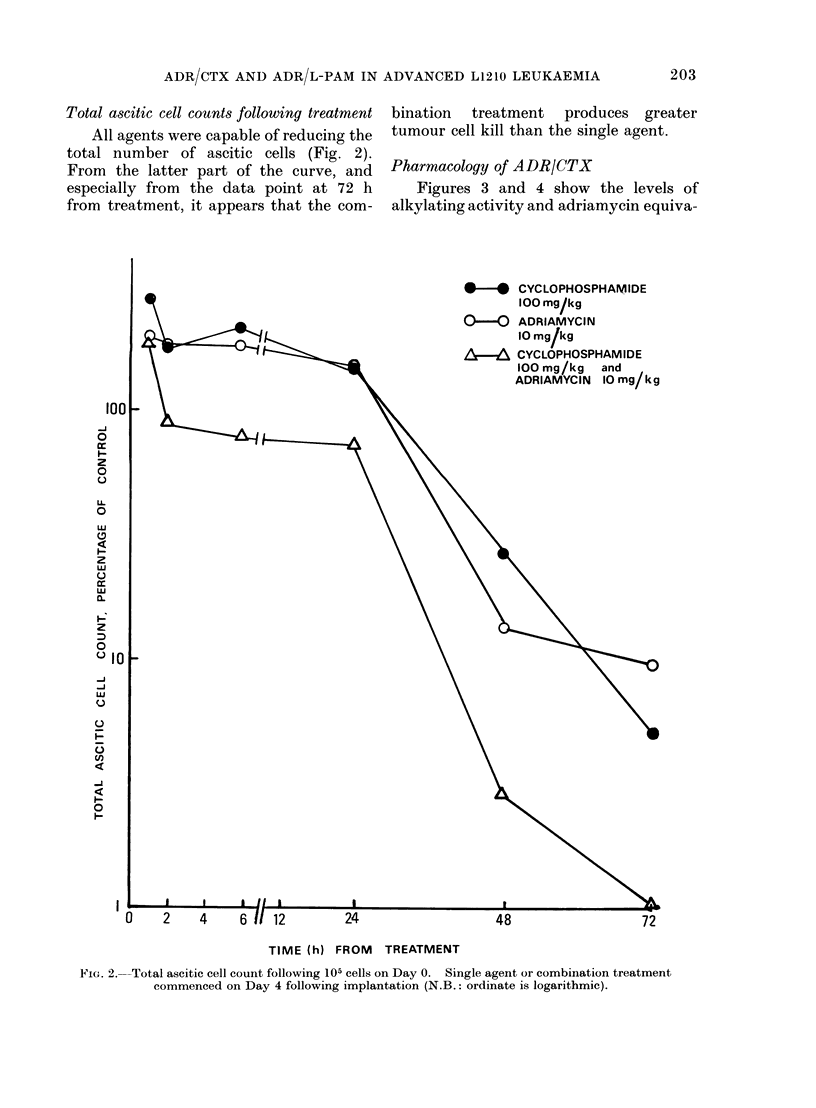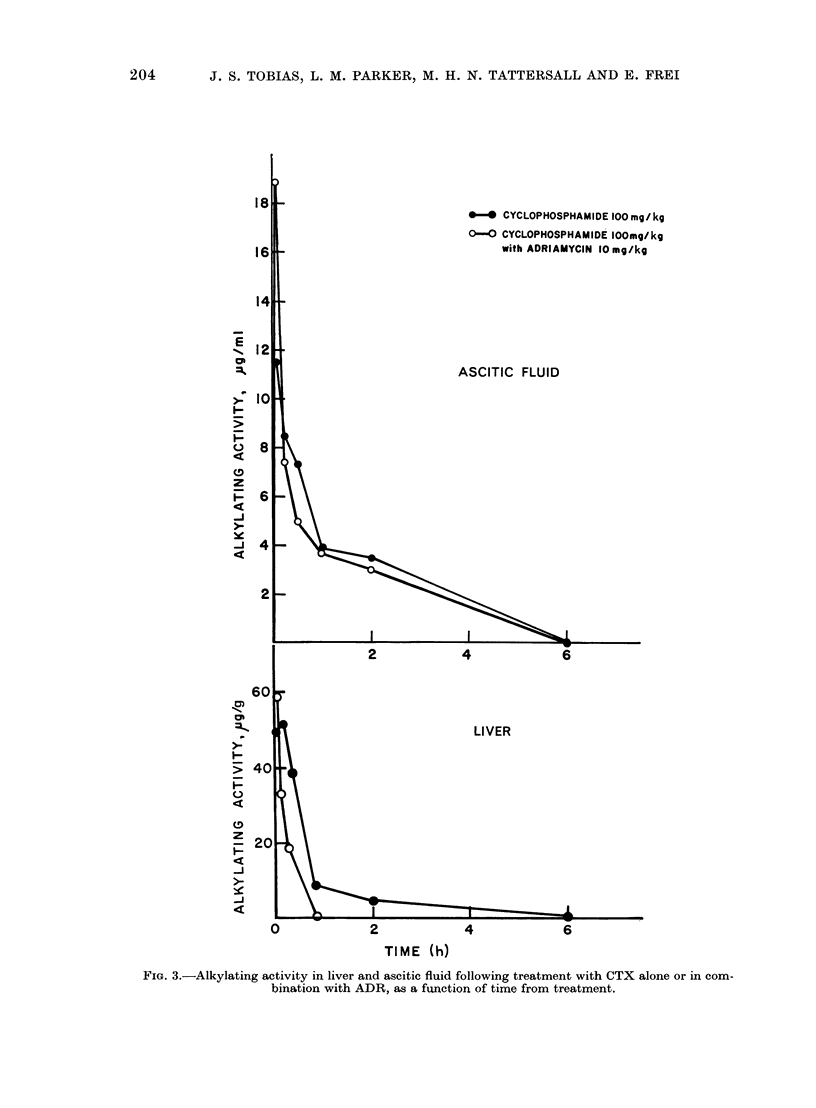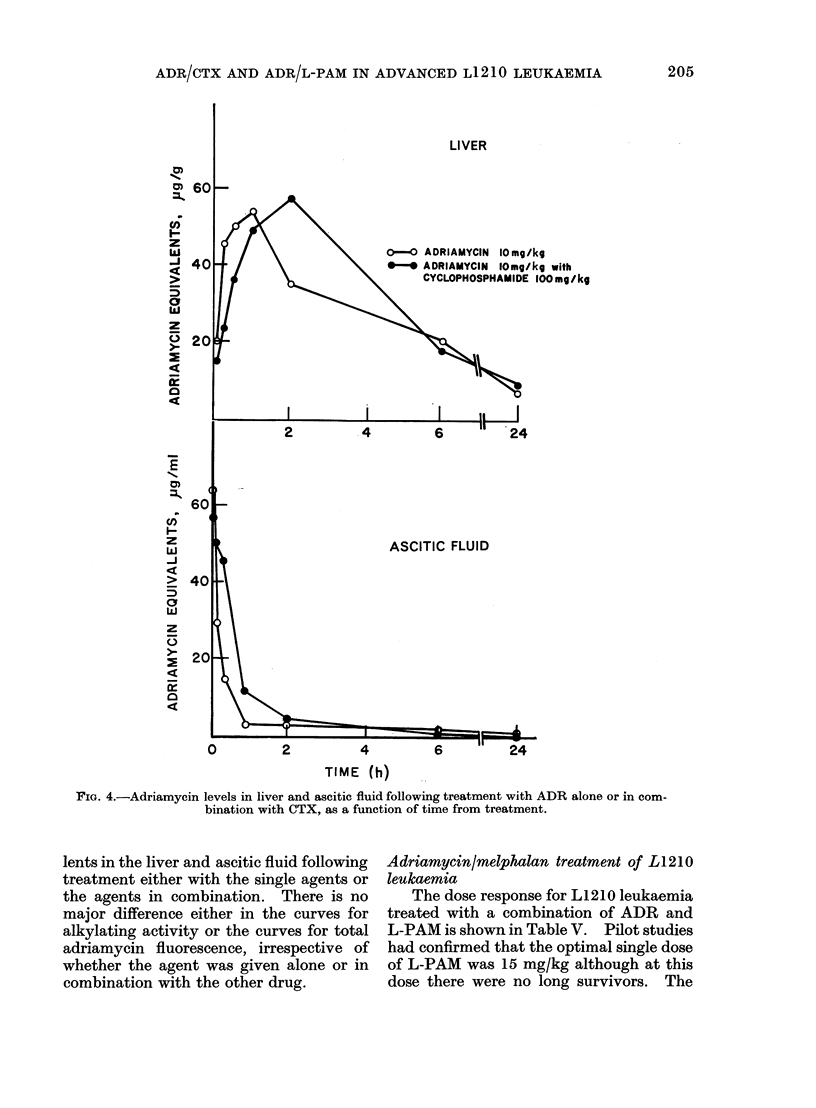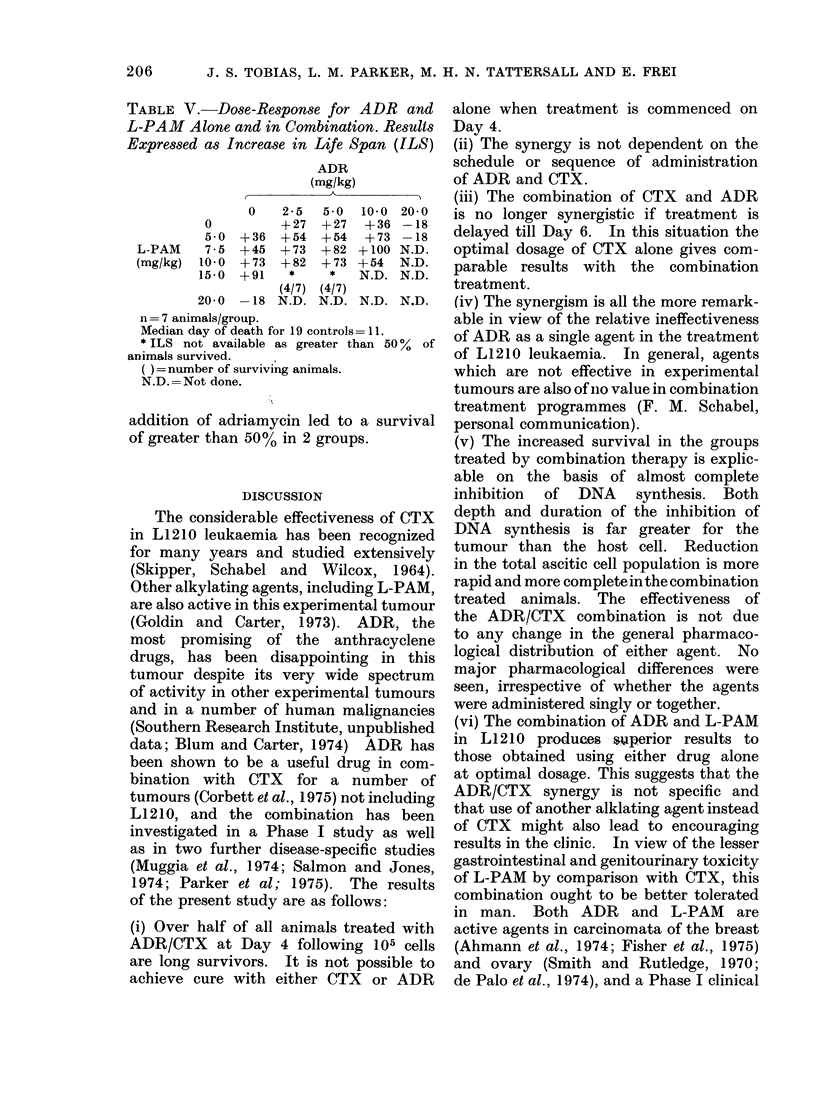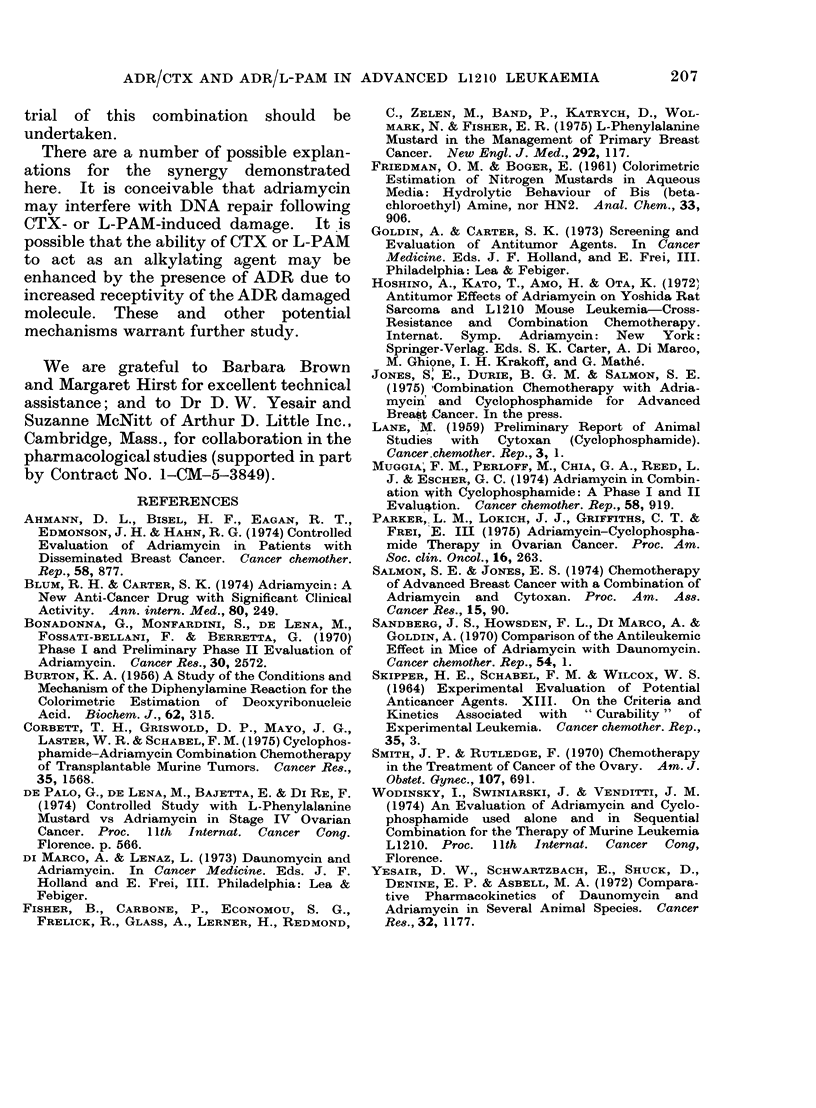# Adriamycin/cyclophosphamide and adriamycin/melphalan in advanced L1210 leukaemia.

**DOI:** 10.1038/bjc.1975.150

**Published:** 1975-08

**Authors:** J. S. Tobias, L. M. Parker, M. H. Tattersall, E. Frei

## Abstract

Adriamycin and cyclophosphamide are active agents in human and experimental tumours. Using the L1210 murine leukaemia, their effectiveness alone and in combination was studied. The combination is highly synergistic in this tumour, resulting in a greater than 50% survival rate when the agents used alone at optimal doses are not curative. DNA synthesis by tumour cells is substantially inhibited and the total ascitic population much reduced. In contrast, DNA synthesis in sensitive host tissues is less disturbed. There is no major difference in the pharmacology of the agents whether given alone or in combination. In very advanced disease the combination is no better than treatment with cyclophosphamide alone. The combination of adriamycin and melphalan in L1210 leukaemia also produces superior results to those obtained using either drug alone at its optimal dosage.


Br. J. Cancer (1975) 32, 199

### ADRIAMYCIN/CYCLOPHOSPHAMIDE AND ADRIAMYCIN/MELPHALAN IN ADVANCED L1210 LEUKAEMIA

J. S. TOBIAS, L. M. PARKER, M. H. N. TATTERSALL* AND E. FREI, III

*From the Sidney Farber Cancer Center, Harvard Medical School, Boston, Massachusetts* 02115, *U.S.A.*

Received 14 April 1975. Accepted 2 May 1975

**Summary.—Adriamycin and cyclophosphamide are active agents in human and experimental tumours. Using the L1210 murine leukaemia, their effectiveness alone and in combination was studied. The combination is highly synergistic in this tumour, resulting in a greater than 50% survival rate when the agents used alone at optimal doses are not curative. DNA synthesis by tumour cells is substantially inhibited and the total ascitic population much reduced. In contrast, DNA synthesis in sensitive host tissues is less disturbed. There is no major difference in the pharmacology of the agents whether given alone or in combination. In very advanced disease the combination is no better than treatment with cyclophosphamide alone. The combination of adriamycin and melphalan in L1210 leukaemia also produces superior results to those obtained using either drug alone at its optimal dosage.**

CYCLOPHOSPHAMIDE (CTX), an alkylating agent with activity against a wide range of experimental and human tumours, has pronounced effects against the L1210 transplantable leukaemia, even in relatively advanced disease (Lane, 1959). L-phenylalanine mustard (Melphalan, L-PAM) is also active in L1210, although this agent has been less thoroughly studied than CTX (Goldin and Carter 1973). More recently, the anthracyclene drugs have been shown to have definite but limited activity against this tumour (Hoshino *et al.*, 1972). Of the anthracyclenes, adriamycin (ADR) is clearly superior to daunorubicin in L1210 and most other experimental tumours (Sandberg *et al.*, 1970; Di Marco and Lenaz, 1973) and in most human malignancies (Bonadonna *et al.*, 1970).

Recent reports on the use of the combination of CTX and ADR have been encouraging, both in human studies (Salmon and Jones, 1974; Muggia *et al.*, 1974; Jones, Durie and Salmon, 1975; Parker *et al.*, 1975), and in several animal systems (Wodinsky, Swiniarski and Venditti, 1974; Corbett *et al.*, 1975).

The purpose of this study was to further evaluate this combination in L1210 leukaemia with respect to (1) the extent of disease, (2) inhibition of normal and leukaemic cell DNA synthesis by the combination, and (3) to investigate the alkylating agent employed.

### MATERIALS AND METHODS

The L1210 leukaemia was obtained from Mr I. Wodinsky, A.D. Little, Inc., Cambridge, Mass. and has been maintained in our laboratories by serial passage. Groups of male $BDF_1$ mice (Jackson Memorial Laboratories, Bar Harbor, Maine) weighing 25–30 g were used. The animals were housed in plastic cages, kept at a constant temperature and provided with water and laboratory chow

* Present address: Charing Cross Hospital, London W6.

Please address reprint requests to: J. S. Tobias, MRCP, The Sidney Farber Cancer Center, 35 Binney Street, Boston, Massachusetts 02115.

Dr Tobias is the recipient of awards from the Cancer Research Campaign and the Mental Health Research Fund, and is the Berkeley Travelling Fellow of the University of Cambridge. Dr Tattersall is supported by the Medical Research Council.

*ad libitum.* Ascitic cell suspensions were prepared in cold sterile saline and all tumour inocula were made in 0·1 ml volumes. All animals were implanted with $10^5$ cells intraperitoneally and treatment was by single intraperitoneal injection on Day 4 following tumour implantation, except for 2 experiments in which treatment was given on Day 6 or Day 8 following implantation. CTX was obtained from Mead, Johnson Laboratories, Evansville, Indiana; L-PAM was obtained from Burroughs Wellcome Company, Research Triangle Park, North Carolina and ADR from Farmitalia, Milan. Solutions were made up in sterile water or ethyl alcohol (L-PAM) and used immediately in all cases.

For the experiments designed to investigate DNA synthesis during treatment, a tritiated thymidine labelling technique was used. Groups of 3 animals were killed by cervical dislocation at various times following treatment. Thirty minutes before killing, tritiated thymidine (specific activity 6·9 Ci/mmol, New England Nuclear, Boston, Mass.) was injected intraperitoneally in a volume of 0·5 ml (total dose 50 μCi/animal). Ascites, small intestine and both femora were removed and DNA extractions were made from these tissues following the method of Burton (1956). The amount of DNA in each sample was read colourimetrically and an aliquot was also added to Aquasol scintillation fluid (New England Nuclear, Boston, Mass.) and read in a Beckman Scintillation counter for 5 min. Results were expressed as disintigrations/min/g of DNA.

Total ascitic cell count was performed in order to confirm that inhibition of DNA synthesis did in fact lead to cell kill, and also to follow the progress of tumour growth during treatment either with a single agent or the combination. The populations of tumour cells were studied using the cytofluorograph and these results will be reported separately.

In order to investigate the tissue distribution of CTX or ADR when given singly or together, a series of experiments was performed in which tissues were extracted at various times following treatment with either a single agent or both drugs in combination. Groups of animals were treated with ADR 10 mg/kg, CTX 100 mg/kg, or a combination of these drugs at the same dosage given by simultaneous intraperitoneal injection. Groups of 3 animals were killed at various times following treatment and the livers and ascitic fluid removed. Ascitic fluid was centrifuged at 4°C and the supernatant retained for analysis. The livers were frozen immediately and maintained in liquid nitrogen until analysis. The tissues were extracted for ADR following the method of Yesair *et al.* (1972) and alkylating activity was measured according to the method of Friedman and Boger (1961), in order to determine the level of cyclophosphamide activity. In all cases appropriate tissue blanks were run.

### RESULTS

### *Treatment of L1210 Leukaemia with ADR and CTX*

The anti-tumour effects of ADR and CTX alone and in combination are shown in Table I, in which the results of 4 separate experiments have been pooled. Long survival was achieved only when the 2 agents were used in combination.

TABLE I.—*Effect of Single Drug and Combination Treatment with ADR or CTX in L1210 Leukaemia (All animals treated on Day 4 only)*

| Treatment (i.p.) | Median day of death | *ILS (*) | Long term survivors |
|---|---|---|---|
| Controls | 11·0 | – | 0/60 |
| CTX 100 mg/kg | 18·5 | 68·0 | 0/20 |
| CTX 200 mg/kg | 20·5 | 86·4 | 0/10 |
| ADR 10 mg/kg | 14·0 | 27·3 | 0/20 |
| ADR 20 mg/kg | 17·0 | 54·5 | 0/10 |
| ADR 10 mg/kg + CTX 100 mg/kg | >200 | – | 25/45 |

*ILS: increased life span.

### *Dose response for ADR and CTX in combination*

The results of a more complete study from ineffectual to toxic dosage of the agents are illustrated in Table II. All animals were treated by single injection on Day 4 only.

### *Schedule dependence of ADR and CTX*

The agents were administered either concurrently or separated by varying time intervals. This was done for a suboptimal

TABLE II.—*Dose-Response for ADR and CTX Alone and in Combination. Results Expressed as Increase in Life Span (ILS)*

| | | ADR (mg/kg) | | | | |
|---|---|---|---|---|---|---|
| | | 0 | 2·5 | 5·0 | 10·0 | 20·0 |
| CTX (mg/kg) | 0 | | +18 | +9 | +36 | +54 |
| | 50 | +27 | +64 | +54 | +91 | −36 |
| | 100 | +54 | +91 | * (8/14) | * (9/14) | −91 |
| | 200 | +73 | +200 | * (8/14) | +100 | −36 |
| | 400 | +18 | +73 | +18 | N.D. | N.D. |

* ILS not available as greater than 50% of animals survived.
( ) = number of surviving animals.
All animals treated by single injection on Day 4 only.
N.D. = Not done.

dosage because the optimal combination dosage produced so many long survivors that small schedule differences would most likely not have been apparent. The results are shown in Table III. There is no clear superiority either of CTX followed by ADR or *vice versa* ($P > 0{\cdot}2$ by *chi* square test).

*Combination treatment in very advanced disease*

The agents were administered either on Day 6 or Day 8 following implantation of tumour in order to test the combination against an increasing tumour burden. The combination treatment is not superior to treatment with an optimal dose of CTX alone although the combination remains superior to treatment with either drug alone at the same dose as is used in the combination. This is shown in Table IV.

*Inhibition of DNA synthesis by ADR/CTX*

The inhibition of DNA synthesis for tumour cells and for host cells *in vivo* is shown in Fig. 1. Pilot studies confirmed that on Day 4 following the implantation of $10^5$ L1210 cells intraperitoneally, the number of leukaemic cells infiltrating the marrow was less than 10%. It is clear that each agent is much more effective and sustained on tumour cells than host cells, even those which are dividing rapidly. Furthermore, the ADR/CTX combination essentially eliminates DNA synthesis in L1210 cells whereas each agent alone allows significant DNA synthesis to continue.

TABLE III.—*The Effect of Drug Separation on CTX/ADR Combinations. Treatment by i.p. Injection on Day 4 Following $10^5$ Cells*

| Treatment (i.p.) | | Median day of death | ILS (%) | Long term survivors |
|---|---|---|---|---|
| Controls | | 11 | – | 0/10 |
| ADR 5 mg/kg | | 14 | 27·2 | 0/10 |
| ADR 10 mg/kg | | 15 | 36·3 | 0/10 |
| CTX 100 mg/kg | | 19 | 72·7 | 0/10 |
| CTX 200 mg/kg | | 20·5 | 86·4 | 0/10 |
| ADR 5 mg/kg | CTX 100 mg/kg h after ADR | | | |
| | +0 | 30 | 173 | 5/14 |
| | +2 | >200 | – | 8/14 |
| | +4 | >200 | – | 7/14 |
| | +6 | >200 | – | 7/14 |
| | +8 | 24·5 | 122 | 3/14 |
| | +24 | 22 | 109 | 2/7 |
| CTX 100 mg/kg | ADR 5 mg/kg h after CTX | | | |
| | +0 | 24·5 | 122 | 5/14 |
| | +2 | >200 | – | 9/14 |
| | +4 | 26 | 136 | 5/14 |
| | +6 | 23 | 109 | 4/14 |
| | +8 | 23·5 | 114 | 2/14 |
| | +24 | 27 | 145 | 5/14 |

TABLE IV.—*The Effect of ADR/CTX in Late Disease*

| | | Day 6 | | Day 8 | |
|---|---|---|---|---|---|
| Treatment | No. of animals | Median day of death | ILS | Median day of death | ILS |
| Controls | 25 | 11 | – | 11 | – |
| ADR 10 mg/kg | 15 | 13 | 18 | 12 | 9 |
| ADR 20 mg/kg | 15 | 16 | 45 | 15 | 36 |
| CTX 100 mg/kg | 15 | 20 | 82 | 20 | 82 |
| CTX 200 mg/kg | 15 | 26 | 136 | 23 | 109 |
| ADR 10 mg/kg & CTX 100 mg/kg | 15 | 24 | 118 | 23 | 109 |

FIG. 1.—Inhibition of DNA synthesis in small intestine (A), normal marrow (B), and L1210 cells (C), by ADR and CTX alone and in combination (ct/min/g DNA, expressed as % control). Note rapid inhibition of tumour DNA synthesis. In normal marrow recovery from CTX-induced inhibition is more rapid than that following ADR-induced inhibition.

*Total ascitic cell counts following treatment*

All agents were capable of reducing the total number of ascitic cells (Fig. 2). From the latter part of the curve, and especially from the data point at 72 h from treatment, it appears that the combination treatment produces greater tumour cell kill than the single agent.

*Pharmacology of ADR/CTX*

Figures 3 and 4 show the levels of alkylating activity and adriamycin equiva-

FIG. 2.—Total ascitic cell count following $10^5$ cells on Day 0. Single agent or combination treatment commenced on Day 4 following implantation (N.B.: ordinate is logarithmic).

FIG. 3.—Alkylating activity in liver and ascitic fluid following treatment with CTX alone or in combination with ADR, as a function of time from treatment.

FIG. 4.—Adriamycin levels in liver and ascitic fluid following treatment with ADR alone or in combination with CTX, as a function of time from treatment.

lents in the liver and ascitic fluid following treatment either with the single agents or the agents in combination. There is no major difference either in the curves for alkylating activity or the curves for total adriamycin fluorescence, irrespective of whether the agent was given alone or in combination with the other drug.

*Adriamycin/melphalan treatment of L1210 leukaemia*

The dose response for L1210 leukaemia treated with a combination of ADR and L-PAM is shown in Table V. Pilot studies had confirmed that the optimal single dose of L-PAM was 15 mg/kg although at this dose there were no long survivors. The

TABLE V.—*Dose-Response for ADR and L-PAM Alone and in Combination. Results Expressed as Increase in Life Span (ILS)*

| | | ADR (mg/kg) | | | | |
|---|---|---|---|---|---|---|
| | | 0 | 2·5 | 5·0 | 10·0 | 20·0 |
| | 0 | | +27 | +27 | +36 | −18 |
| | 5·0 | +36 | +54 | +54 | +73 | −18 |
| L-PAM | 7·5 | +45 | +73 | +82 | +100 | N.D. |
| (mg/kg) | 10·0 | +73 | +82 | +73 | +54 | N.D. |
| | 15·0 | +91 | * (4/7) | * (4/7) | N.D. | N.D. |
| | 20·0 | −18 | N.D. | N.D. | N.D. | N.D. |

n = 7 animals/group.
Median day of death for 19 controls = 11.
* ILS not available as greater than 50% of animals survived.
( ) = number of surviving animals.
N.D. = Not done.

addition of adriamycin led to a survival of greater than 50% in 2 groups.

### DISCUSSION

The considerable effectiveness of CTX in L1210 leukaemia has been recognized for many years and studied extensively (Skipper, Schabel and Wilcox, 1964). Other alkylating agents, including L-PAM, are also active in this experimental tumour (Goldin and Carter, 1973). ADR, the most promising of the anthracyclene drugs, has been disappointing in this tumour despite its very wide spectrum of activity in other experimental tumours and in a number of human malignancies (Southern Research Institute, unpublished data; Blum and Carter, 1974) ADR has been shown to be a useful drug in combination with CTX for a number of tumours (Corbett *et al.*, 1975) not including L1210, and the combination has been investigated in a Phase I study as well as in two further disease-specific studies (Muggia *et al.*, 1974; Salmon and Jones, 1974; Parker *et al;* 1975). The results of the present study are as follows:

(i) Over half of all animals treated with ADR/CTX at Day 4 following $10^5$ cells are long survivors. It is not possible to achieve cure with either CTX or ADR alone when treatment is commenced on Day 4.

(ii) The synergy is not dependent on the schedule or sequence of administration of ADR and CTX.

(iii) The combination of CTX and ADR is no longer synergistic if treatment is delayed till Day 6. In this situation the optimal dosage of CTX alone gives comparable results with the combination treatment.

(iv) The synergism is all the more remarkable in view of the relative ineffectiveness of ADR as a single agent in the treatment of L1210 leukaemia. In general, agents which are not effective in experimental tumours are also of no value in combination treatment programmes (F. M. Schabel, personal communication).

(v) The increased survival in the groups treated by combination therapy is explicable on the basis of almost complete inhibition of DNA synthesis. Both depth and duration of the inhibition of DNA synthesis is far greater for the tumour than the host cell. Reduction in the total ascitic cell population is more rapid and more complete in the combination treated animals. The effectiveness of the ADR/CTX combination is not due to any change in the general pharmacological distribution of either agent. No major pharmacological differences were seen, irrespective of whether the agents were administered singly or together.

(vi) The combination of ADR and L-PAM in L1210 produces superior results to those obtained using either drug alone at optimal dosage. This suggests that the ADR/CTX synergy is not specific and that use of another alklating agent instead of CTX might also lead to encouraging results in the clinic. In view of the lesser gastrointestinal and genitourinary toxicity of L-PAM by comparison with CTX, this combination ought to be better tolerated in man. Both ADR and L-PAM are active agents in carcinomata of the breast (Ahmann *et al.*, 1974; Fisher *et al.*, 1975) and ovary (Smith and Rutledge, 1970; de Palo *et al.*, 1974), and a Phase I clinical trial of this combination should be undertaken.

There are a number of possible explanations for the synergy demonstrated here. It is conceivable that adriamycin may interfere with DNA repair following CTX- or L-PAM-induced damage. It is possible that the ability of CTX or L-PAM to act as an alkylating agent may be enhanced by the presence of ADR due to increased receptivity of the ADR damaged molecule. These and other potential mechanisms warrant further study.

We are grateful to Barbara Brown and Margaret Hirst for excellent technical assistance; and to Dr D. W. Yesair and Suzanne McNitt of Arthur D. Little Inc., Cambridge, Mass., for collaboration in the pharmacological studies (supported in part by Contract No. 1–CM–5–3849).

REFERENCES

AHMANN, D. L., BISEL, H. F., EAGAN, R. T., EDMONSON, J. H. & HAHN, R. G. (1974) Controlled Evaluation of Adriamycin in Patients with Disseminated Breast Cancer. *Cancer chemother. Rep.*, **58**, 877.

BLUM, R. H. & CARTER, S. K. (1974) Adriamycin: A New Anti-Cancer Drug with Significant Clinical Activity. *Ann. intern. Med.*, **80**, 249.

BONADONNA, G., MONFARDINI, S., DE LENA, M., FOSSATI-BELLANI, F. & BERRETTA, G. (1970) Phase I and Preliminary Phase II Evaluation of Adriamycin. *Cancer Res.*, **30**, 2572.

BURTON, K. A. (1956) A Study of the Conditions and Mechanism of the Diphenylamine Reaction for the Colorimetric Estimation of Deoxyribonucleic Acid. *Biochem. J.*, **62**, 315.

CORBETT, T. H., GRISWOLD, D. P., MAYO, J. G., LASTER, W. R. & SCHABEL, F. M. (1975) Cyclophosphamide–Adriamycin Combination Chemotherapy of Transplantable Murine Tumors. *Cancer Res.*, **35**, 1568.

DE PALO, G., DE LENA, M., BAJETTA, E. & DI RE, F. (1974) Controlled Study with L-Phenylalanine Mustard vs Adriamycin in Stage IV Ovarian Cancer. *Proc. 11th Internat. Cancer Cong.* Florence. p. 566.

DI MARCO, A. & LENAZ, L. (1973) Daunomycin and Adriamycin. In *Cancer Medicine*. Eds. J. F. Holland and E. Frei, III. Philadelphia: Lea & Febiger.

FISHER, B., CARBONE, P., ECONOMOU, S. G., FRELICK, R., GLASS, A., LERNER, H., REDMOND, C., ZELEN, M., BAND, P., KATRYCH, D., WOLMARK, N. & FISHER, E. R. (1975) L-Phenylalanine Mustard in the Management of Primary Breast Cancer. *New Engl. J. Med.*, **292**, 117.

FRIEDMAN, O. M. & BOGER, E. (1961) Colorimetric Estimation of Nitrogen Mustards in Aqueous Media: Hydrolytic Behaviour of Bis (beta-chloroethyl) Amine, nor HN2. *Anal. Chem.*, **33**, 906.

GOLDIN, A. & CARTER, S. K. (1973) Screening and Evaluation of Antitumor Agents. In *Cancer Medicine*. Eds. J. F. Holland, and E. Frei, III. Philadelphia: Lea & Febiger.

HOSHINO, A., KATO, T., AMO, H. & OTA, K. (1972) Antitumor Effects of Adriamycin on Yoshida Rat Sarcoma and L1210 Mouse Leukemia—Cross-Resistance and Combination Chemotherapy. Internat. Symp. Adriamycin: New York: Springer-Verlag. Eds. S. K. Carter, A. Di Marco, M. Ghione, I. H. Krakoff, and G. Mathé.

JONES, S. E., DURIE, B. G. M. & SALMON, S. E. (1975) Combination Chemotherapy with Adriamycin and Cyclophosphamide for Advanced Breast Cancer. In the press.

LANE, M. (1959) Preliminary Report of Animal Studies with Cytoxan (Cyclophosphamide). *Cancer chemother. Rep.*, **3**, 1.

MUGGIA, F. M., PERLOFF, M., CHIA, G. A., REED, L. J. & ESCHER, G. C. (1974) Adriamycin in Combination with Cyclophosphamide: A Phase I and II Evaluation. *Cancer chemother. Rep.*, **58**, 919.

PARKER, L. M., LOKICH, J. J., GRIFFITHS, C. T. & FREI, E. III (1975) Adriamycin–Cyclophosphamide Therapy in Ovarian Cancer. *Proc. Am. Soc. clin. Oncol.*, **16**, 263.

SALMON, S. E. & JONES, E. S. (1974) Chemotherapy of Advanced Breast Cancer with a Combination of Adriamycin and Cytoxan. *Proc. Am. Ass. Cancer Res.*, **15**, 90.

SANDBERG, J. S., HOWSDEN, F. L., DI MARCO, A. & GOLDIN, A. (1970) Comparison of the Antileukemic Effect in Mice of Adriamycin with Daunomycin. *Cancer chemother. Rep.*, **54**, 1.

SKIPPER, H. E., SCHABEL, F. M. & WILCOX, W. S. (1964) Experimental Evaluation of Potential Anticancer Agents. XIII. On the Criteria and Kinetics Associated with "Curability" of Experimental Leukemia. *Cancer chemother. Rep.*, **35**, 3.

SMITH, J. P. & RUTLEDGE, F. (1970) Chemotherapy in the Treatment of Cancer of the Ovary. *Am. J. Obstet. Gynec.*, **107**, 691.

WODINSKY, I., SWINIARSKI, J. & VENDITTI, J. M. (1974) An Evaluation of Adriamycin and Cyclophosphamide used alone and in Sequential Combination for the Therapy of Murine Leukemia L1210. *Proc. 11th Internat. Cancer Cong*, Florence.

YESAIR, D. W., SCHWARTZBACH, E., SHUCK, D., DENINE, E. P. & ASBELL, M. A. (1972) Comparative Pharmacokinetics of Daunomycin and Adriamycin in Several Animal Species. *Cancer Res.*, **32**, 1177.